# Tks5 Regulates Synaptic Podosome Formation and Stabilization of the Postsynaptic Machinery at the Neuromuscular Junction

**DOI:** 10.3390/ijms222112051

**Published:** 2021-11-07

**Authors:** Marcin Pęziński, Kamila Maliszewska-Olejniczak, Patrycja Daszczuk, Paula Mazurek, Paweł Niewiadomski, Maria Jolanta Rędowicz

**Affiliations:** 1 Laboratory of Synaptogenesis, Nencki Institute of Experimental Biology, Polish Academy of Sciences, 3 Pasteur St., 02-787 Warszawa, Poland; kamila_maliszewska-olejniczak@sggw.edu.pl (K.M.-O.); p.daszczuk@cent.uw.edu.pl (P.D.); p.mazurek@cent.uw.edu.pl (P.M.); p.niewiadomski@cent.uw.edu.pl (P.N.); 2 Laboratory of Molecular Basis of Cell Motility, Nencki Institute of Experimental Biology, Polish Academy of Sciences, 3 Pasteur St., 02-787 Warszawa, Poland; j.redowicz@nencki.edu.pl; 3 Centre of New Technologies, Laboratory of Molecular and Cellular Signalling, University of Warsaw, 2c Stefan Banach St., 02-787 Warszawa, Poland

**Keywords:** actin, neuromuscular junction, podosomes, postsynaptic machinery, rapsyn, Tks5

## Abstract

Currently, the etiology of many neuromuscular disorders remains unknown. Many of them are characterized by aberrations in the maturation of the neuromuscular junction (NMJ) postsynaptic machinery. Unfortunately, the molecular factors involved in this process are still largely unknown, which poses a great challenge for identifying potential therapeutic targets. Here, we identified Tks5 as a novel interactor of αdystrobrevin-1, which is a crucial component of the NMJ postsynaptic machinery. Tks5 has been previously shown in cancer cells to be an important regulator of actin-rich structures known as invadosomes. However, a role of this scaffold protein at a synapse has never been studied. We show that Tks5 is crucial for remodeling of the NMJ postsynaptic machinery by regulating the organization of structures similar to the invadosomes, known as synaptic podosomes. Additionally, it is involved in the maintenance of the integrity of acetylcholine receptor (AChR) clusters and regulation of their turnover. Lastly, our data indicate that these Tks5 functions may be mediated by its involvement in recruitment of actin filaments to the postsynaptic machinery. Collectively, we show for the first time that the Tks5 protein is involved in regulation of the postsynaptic machinery.

## 1. Introduction

The neuromuscular junction (NMJ) is a complex system composed of a motor nerve terminal that constitutes the presynaptic site, muscle postsynaptic machinery, and perisynaptic Schwann cells. The main function of this synapse, i.e., transmission of the signals inducing muscle contraction, is regulated by a plethora of molecular processes that contribute to maturation and maintenance of the junction, ensuring that the synaptic transmission will be undisturbed [1,2]. Unfortunately, the regulators of many of these molecular events still remain undiscovered, which is reflected by the fact that a great number of identified neuromuscular diseases are of unknown etiology [3]. For this reason, studies that aim at identification of new factors involved in regulation of the formation and development of NMJ are of vital importance.

During the development, clusters of acetylcholine receptors (AChRs) increase in size and density, but also undergo significant remodeling of their topology [4]. At the early stages they assume an oval, plaque shape, which becomes perforated during maturation assuming a more complex, pretzel-like morphology [5]. While the purpose of such rearrangement is not understood, a failure to undergo through this process very often correlates to neuromuscular disorders [6]. Interestingly, the pretzel-like shape of a mature postsynaptic machinery encompasses the motor nerve ending so that the AChRs localize directly below the presynaptic terminal. The areas between AChR branches harbor a number of proteins that are involved in adhesion and stabilization of the apparatus, such as nestin [7,8]. The fully developed postsynaptic machinery is made up of functional domains that are either directly involved in neurotransmission (such as the AChR-rich branches) or play supportive roles (the AChR-poor areas between the branches) [8]. Importantly, the postsynaptic machinery in the in vitro cultured myotubes also has distinguishable macrodomains that elicit different functions. A good example are the synaptic podosomes, present in the AChR-poor areas of the cluster, which form a large synaptic domain enriched with actin and proteins involved in cell adhesion [9]. It was recently demonstrated that synaptic podosomes capable of degrading the extracellular matrix (ECM) are involved in remodeling of AChR clusters in laminin-cultured myotubes [9,10,11,12]. It was the first time that these organelles have been shown to play a role in the biology of a synapse; however, a complete evidence for their existence in vivo is still lacking. So far, several known podosomal markers have been identified to be present in the perforations of a mature NMJ, including Amotl2 and pleckstrin homology-like domain family B member 2 (LL5B), suggesting that a similar remodeling mechanism might occur in vivo [10,13,14].

The dystrophin-associated glycoprotein complex (DGC) is a large protein complex that harbors multiple essential components of the postsynaptic machinery and stabilizes AChRs within the membrane by interacting both with extracellular matrix proteins such as laminins and the cytoskeleton [15,16,17]. One of the crucial constituents of the DGC is αdystrobrevin-1 (αDB1). Aberrations in alternative splicing of this protein are associated with myotonic dystrophy type 1 (DM1) in humans, and knockout mice have unstable AChR clusters with aberrant shapes [18,19]. In order to elicit the function of αDB1, our laboratory has previously identified a number of its interacting partners. Among those were proteins such as Rho guanine nucleotide exchange factor 5 (Arhgef5), growth factor-receptor bound protein 2 (Grb2) and liprin-α-1 [20,21,22]. All of them are involved in regulation of the muscle postsynaptic machinery, highlighting the importance of αDB1 in synapse organization. Many molecular functions of αDB1 are attributed to the three phosphorylatable tyrosine residues located in its C-terminus, phosphorylation of which are critical for rescuing knockout phenotypes [23,24]. For this reason, we performed a screen for proteins that specifically interact with either phosphorylated or unphosphorylated tyrosine 730 of αDB1. The mass spectrometry analysis revealed that Tks5 protein binds specifically to unphosphorylated αDB1. Previously, it has been shown that Tks5 is enriched at the NMJ postsynaptic machinery formed by laminin-cultured myotubes and localizes to synaptic podosomes in vitro [9]. Tks5 is a scaffold protein that is a marker of podosomal structures in cancer and other cell types [25,26]. It is also regulated by proto-oncogene non-receptor tyrosine-protein kinase Src (Src kinase) at early stages of podosome formation pathway [25,27]. Its function in skeletal muscles, however, remains unknown. Similarly, the localization and function of Tks5 at the NMJ (or any other synapses) have never been studied. Based on the recent findings that postsynaptic machinery in vitro undergoes developmental remodeling via synaptic podosomes and the fact that maturation of a synapse is often compromised in various neuromuscular disorders, Tks5 could be an important regulator of postsynaptic maturation.

Here, we present evidence that Tks5 is indeed important for synaptic podosome formation as its depletion influences the organization of AChRs within the clusters, possibly through its involvement in recruitment of the actin cytoskeleton to the postsynaptic apparatus. Moreover, this is the first time when Tks5 protein was shown to be involved in regulation of organization of the postsynaptic machinery.

## 2. Results

### 2.1. Tks5 Interacts with Unphosphorylated Adystrobrevin-1 and Localizes to the NMJ

**Figure 1:**
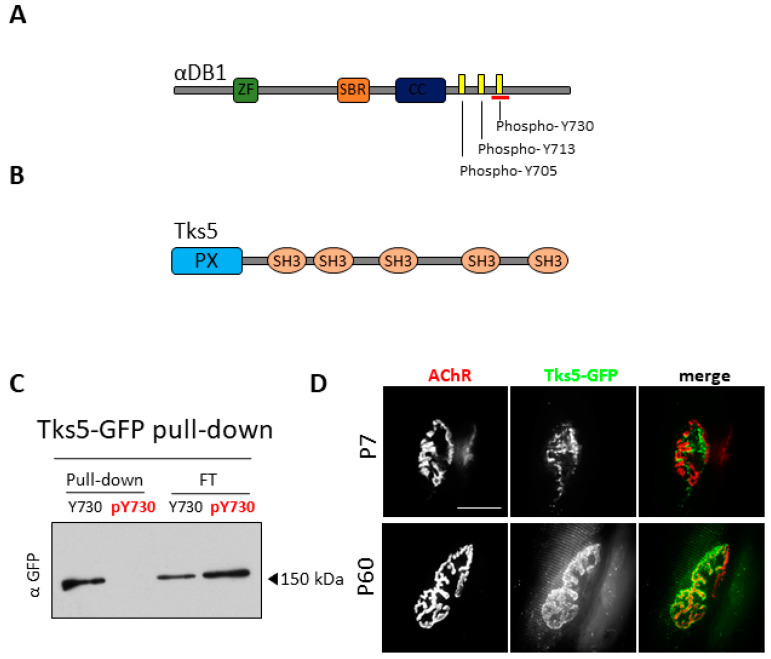
Tks5 interacts with α-dystrobrevin 1. (**A**) Schematic representation of αDB1 domain organization and location of phosphorylation sites. The red line represents the fragment of the protein corresponding to the peptides used in experiments. ZF—zinc finger domain; SBR—syntrophin binding region; CC—coiled-coil domain. (**B**) Schematic representation of Tks5 protein domain organization. PX—phox homology domain; SH3—src homology 3 domain. The PX domain is responsible for targeting the protein to the plasma membrane, while the SH3 domains are responsible for binding numerous proteins, including actin regulators. (**C**) Tks-GFP pull-down using αDB1 Y730 peptides. HEK cells were transfected with Tks5-GFP construct followed by the lysis and loading of the cell lysates on beads coated with phosphorylated and unphosphorylated αDB1 peptide Y730. FT—flow-through; Pull-down—eluted sample; pY730—phosphorylated αDB1 peptide; Y730—unphosphorylated αDB1 peptide. (**D**) Tks5 localizes to AChR-poor areas of the NMJ in early development (P7) and to AChR-rich branches in adulthood (P60). Tks5-GFP constructs were electroporated into TA muscle and visualized 7 days later. The muscles were also stained with BTX-Alexa-555 to visualize the AChRs. Scale bar, 10 µm.

αDB1 has three potential phosphorylatable tyrosine residues: Y705, Y713, and Y730, located at its C-terminus (Figure 1A). Since these sites are likely to be involved in regulating the function of this protein, it is worthwhile to investigate the interactors that bind to these tyrosine residue(s) in the phosphorylation-dependent manner [23]. To identify proteins that interact with αDB1, we performed a pulldown experiment using magnetic beads coated with either unphosphorylated or phosphorylated αDB1 peptide containing tyrosine Y730 (unphosphorylated and phosphorylated, Y730 and pY730, respectively) as a bait to pull down its interacting partners from a lysate of C2C12 myotubes. The eluted samples were then subjected to a mass spectrometry analysis. Through this, we identified, among others, Tks5 (a 124-kDa scaffold protein with five SH3 domains) as a novel interactor of unphosphorylated (but not of phosphorylated) αDB1 Y730 peptide (Figure 1B). The mode of Tks5 interaction with αDB1 was confirmed through a pulldown assay, where lysates of Tks5-GFP-transfected HEK cells were incubated with magnetic beads coated with the same αDB1 peptides that were used for mass spectrometry analysis (Figure 1C).

In order to see whether Tks5 localizes to the NMJ in vivo, we electroporated tibialis anterior (TA) muscles at postnatal day 7 (P7) and P60 wild-type mice with Tks5-GFP constructs and evaluated the AChR-associated fluorescence signals under confocal microscope (Figure 1D). The postsynaptic machinery in P7 mice is still immature and its morphology differs from that of P60 mice, which are considered to have fully developed AChR clusters. The receptors were visualized with fluorescently labeled α-bungarotoxin (BTX), which is a venom toxin specifically binding to AChRs. Interestingly, the analysis revealed that localization of Tks5 changes during the early postnatal period. In P7 mice, the Tks5-GFP was present predominantly within the AChR-poor areas of the postsynaptic machinery (top panel). On the other hand, the GFP signal in P60 mice was predominantly localized to the AChR-rich branches of the NMJ (bottom panel). This suggests that Tks5 may have a function at the NMJ and that this function may change during the development.

### 2.2. Tks5 Is Essential for Synaptic Podosome Formation

**Figure 2:**
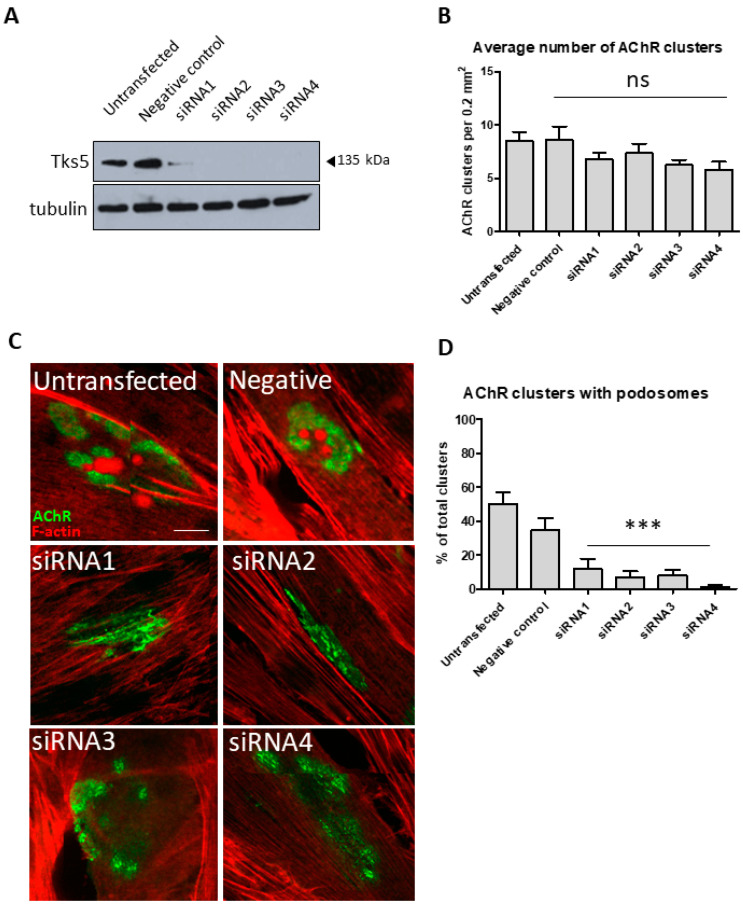
Tks5 affects the formation of synaptic podosomes. (**A**) Western blot analysis of Tks5 protein in lysates of C2C12 myoblasts treated with either unspecific (negative control) or Tks5 siRNAs. Tubulin was used as a loading control. (**B**) Measurement of the number of clusters found in C2C12 myotubes in control and Tks5 KD cells. The data represent the average number of clusters found in 12 different 0.2-mm^2^ fields. ns= not significant. (**C**) Example images of AChR clusters in control and Tks5-depleted C2C12 myotubes lacking synaptic podosomes. AChRs were visualized with BTX-Alexa-488 and actin with Alexa555-phalloidin. (**D**) Quantification of the effect of Tks5 KD on formation of synaptic podosomes. Graph represents the percentages of clusters containing synaptic podosomes in control cells and those treated with the indicated siRNAs. One-way ANOVA with Dunnett test (untransfected control used as reference) was used to determine the statistical significance. Forty AChR clusters were used per point in the analysis. Error bars represent SEM. *** *p* < 0.0001. Scale bar, 10 μm.

In order to investigate a potential role of Tks5 (a known actin-regulator) in terms of synaptic regulation, we performed an siRNA-mediated knockdown of this protein in C2C12 myotubes using four different siRNAs (Figure 2) [28]. Two types of control were used, untransfected cells and cells transfected, with an unspecific, scrambled siRNA sequence, denoted as a negative control. Substantial depletion of Tks5 protein in the lysates of these cells was observed for all four siRNA sequences (Figure 2A). Staining for AChR revealed that the number of AChR clusters in C2C12 myotubes was unchanged upon silencing of the Tks5 expression (Figure 2B). However, Tks5 KD resulted in a nearly complete disappearance of synaptic podosomes (Figure 2C,D). The clusters still underwent certain remodeling, as they did not appear as simple plaques, which are considered to be the first developmental stage of the AChR cluster, but as dispersed assemblies. These observations suggest that Tks5 could be specifically involved in a podosome-driven remodeling of AChR cluster topology, and other pathways involved in this process are likely unaffected by the silencing of the expression of this protein. This is also consistent with the localization of Tks5-GFP to the AChR-poor areas in P7 mice, as it has been shown before that numerous proteins involved in podosome formation are found within those domains [10].

### 2.3. Absence of Tks5 Leads to Instability of AChR Clusters and Compromised Turnover of the Receptors

**Figure 3:**
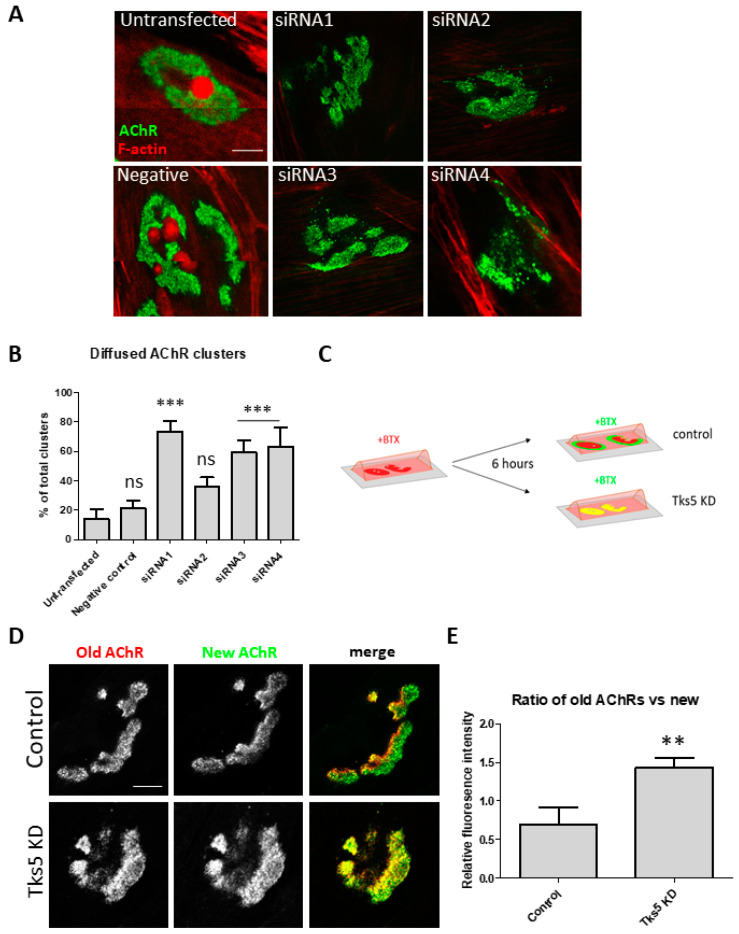
AChR clusters become fragmented after depletion of Tks5 and exhibit compromised receptor turnover. (**A**) Examples of AChR clusters in control cells and after treatment with siRNA against Tks5 exhibiting fragmentation of the receptor clusters. AChRs were visualized with BTX-Alexa-488 and actin with Alexa555-phalloidin. (**B**) Quantification of the effect of Tks5 KD. The graph represents the percentages of fragmented clusters in control cells and those treated with the indicated siRNAs. One-way ANOVA with Dunnett test (untransfected control used as reference) were used to determine the statistical significance. Thirty AChR clusters were used per point in the analysis. ns= not significant. (**C**) Schematic diagram showing the experimental design for panels D and E. Briefly, BTX-Alexa-555 was added live to C2C12 myotubes for 10 min and washed away. After 6 h the cells were fixed and BTX-Alexa-488 was used to label the newly deposited receptors. (**D**) Example AChR clusters after transfection with siRNA1. Staining as described in C. New receptors were added peripherally to the pre-existing clusters in control cells; however, after Tks5 knockdown the two signals overlap. (**E**) Quantification of the fluorescence intensity shown as ratio of old AChRs to new ones. Unpaired t test was used to determine the statistical significance. 30 AChR clusters were used per point in the analysis. Error bars represent SEM. ** *p* < 0.001; *** *p* < 0.0001. Scale bar, 10 μm.

Besides the observed effect on podosome formation, we also noticed that Tks5 knockdown in C2C12 myotubes caused apparent instability of the AChR clusters, represented by fragmentation of the postsynaptic machinery. All except one of the tested siRNAs elicited this effect (Figure 3A,B). Similar aberrations are observed at the postsynaptic machinery in vivo when the proteins constituting DGC, such as for example dystrophin are silenced [29]. While the absence of synaptic podosomes and significant fragmentation of the AChR clusters are prominent phenotypes of C2C12 in the absence of Tks5, we went on to investigate whether dynamics of the postsynaptic machinery could be affected by the lack of this protein. For this, we incubated the cells with BTX-Alexa-555 for 10 min to label the pre-existing clusters, and after 6 h BTX-Alexa-488 to visualize the newly added receptors (Figure 3C). As a result, we observed that while in the Tks5-depleted cells the new receptors (stained in green) have been added to the clusters, they did so in an unorganized manner, randomly inserting themselves in the entire cluster area (Figure 3D). This is in contrast to control conditions, where new receptors are added circumferentially to the pre-existing assemblies as described before [30]. Additionally, analysis of the ratio of the fluorescence intensity of old receptors to the new ones revealed that after Tks5 knockdown significantly fewer receptors were added to the pre-existing clusters (Figure 3E).

### 2.4. Tks5 Is Required for Association of Actin with the Postsynaptic Machinery

**Figure 4:**
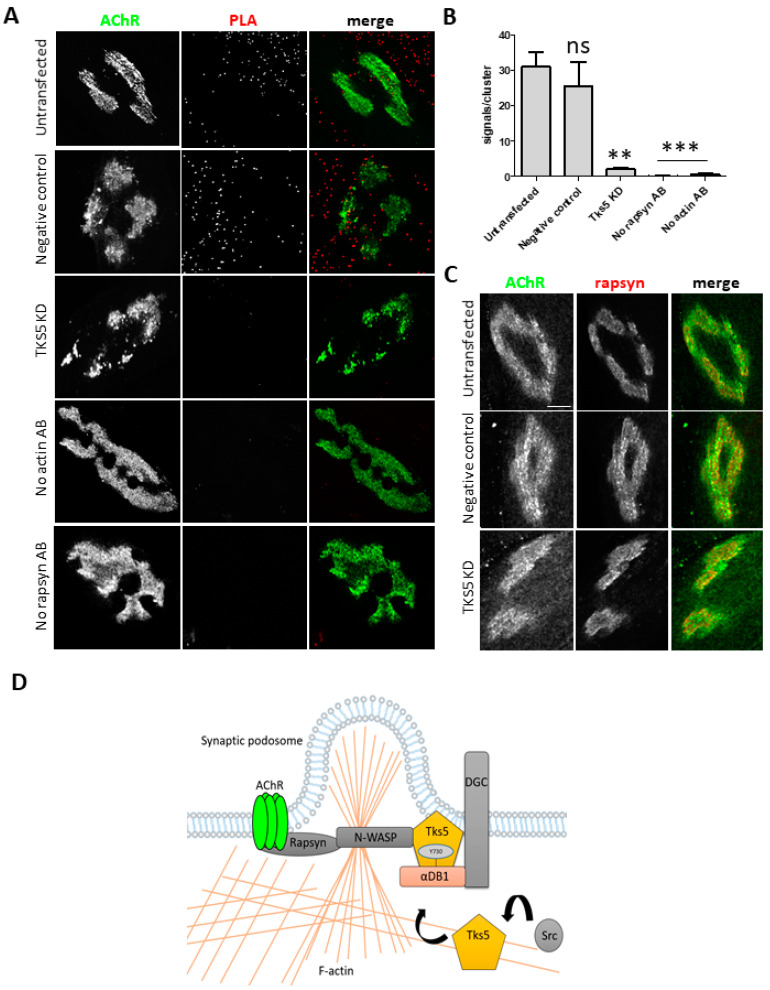
Tks5 is needed for associating the postsynaptic machinery with actin. (**A**) Proximity ligation assay between actin and rapsyn in C2C12 myotubes. Each red punctum denotes a site of interaction between actin and rapsyn molecules. Cells where Tks5 was knocked down had a dramatic decrease in the amount of these interactions. Similarly, no PLA signals were detected when only one primary antibody was used, confirming the specificity of the method. (**B**) Quantification of PLA signals. One-way ANOVA with Dunnett test (untransfected control used as reference) were used to determine the statistical significance. 20 AChR clusters were used per point in the analysis. ns= not significant. (**C**) Rapsyn localization is not affected by the knock down of Tks5. AChRs were visualized with BTX-Alexa-488. (**D**) Schematic representation of proposed mode of Tks5 action at the postsynaptic machinery. Likely, after c-SRC phosphorylates Tks5 it gets recruited to αDB1. Tks5 is likely recruited to αDB1 after phosphorylation by c-SRC. This triggers the association of Tks5 with N-WASP and other actin organizers that are involved in formation of synaptic podosomes. At the same time, actin filaments recruited by Tks5 through this mechanism could be used for delivery of new AChRs. Error bars represent SEM. *** *p* < 0.0001; ** *p* < 0.001. Scale bar, 10 μm.

Lack of synaptic podosomes, their significant fragmentation, and compromised maintenance mechanisms of these assemblies, as well as known association of Tks5 with the actin cytoskeleton indicate that the observed Tks5-associated aberrations could be a result of impairment in the actin cytoskeleton organization. To see how the absence of Tks5 influences interaction of actin filaments with proteins associated with the AChRs, we performed a proximity ligation assay (PLA), which detects interactions between two proteins located within 20–40 nm from each other (Figure 4). One of the AChR-associated proteins at the postsynaptic machinery is rapsyn, which anchors AChRs to the cortical actin cytoskeleton [31]. The PLA assay revealed a substantial decrease in the number of actin-rapsyn interactions when Tks5 was absent (Figure 4A,B), suggesting that this protein is indeed required for association of actin filaments with the constituents of the postsynaptic machinery. This could explain the phenotypes described above (Figure 2 and Figure 3). Importantly, rapsyn localization was unaffected by the absence of Tks5, as it colocalized with the AChRs to a similar degree as in control cells (Figure 4C).

## 3. Discussion

Aberrant maturation of the NMJ is a characteristic for many neuromuscular disorders; however, the underlying mechanisms for such changes are still not entirely understood [32]. The recent discovery that remodeling of the postsynaptic machinery clusters in laminin cultured myotubes is driven by synaptic podosomes, actin-rich structures that are able to invade ECM and implicated in cellular adhesion, has opened up doors for investigating actin regulators and their role(s) in postsynaptic maturation [9,13]. Our mass spectrometry analysis of αDB1 interactors revealed Tks5 as a protein that interacts with unphosphorylated form of αDB1, a critical component of the DGC. Tks5 (formerly known as FISH) is an adaptor/scaffold protein for crucial actin organizers such as N-WASP [33]. It is also regulated by proto-oncogene non-receptor tyrosine-protein kinase Src (Src kinase) at early stages of podosome formation pathway [25,27]. Podosomes have also been described in multiple cell types including macrophages, dendritic cells, endothelial cells, vascular smooth muscle cells, osteoclasts, fibroblasts, and cancer cells [34]. Tks5 has been studied in the context of cancer metastasis, where it regulates formation of invadopodia, the structures analogous to podosomes in non-cancer cells, which support cancer spreading [25,35]. Importantly, Tks5 also interacts with the ADAM family of metalloproteases (disintegrates), and with the dystroglycan complex (Seals, Azucena et al. 2005) [27]. However, a role of Tks5 at the muscle synapse has never been extensively studied.

Our data revealed for the first time that silencing of Tks5 expression in C2C12 myotubes led to a number of phenotypes. First, almost a complete disappearance of synaptic podosomes was observed (Figure 2). Second, compromised integrity of AChR clusters characterized by a significant fragmentation or dispersion of the assembly similar to the one observed in neuromuscular disease models such as mdx mice was visible (Figure 3). Third, new receptors added to the pre-existing clusters were not localized to the cluster periphery (as observed for control cells), but instead were randomly distributed throughout the cluster assembly. Importantly, when we measured the fluorescence signal from the newly added receptors it turned out that the receptor turnover was greatly reduced in the absence of Tks5 (Figure 3).

Podosomes have only very recently been proposed to function in regulation of postsynaptic machinery remodeling. The mechanisms that drive this process are largely unknown despite its relevance in various pathological states of the NMJ [32]. Our data suggests that Tks5 is an important podosomal regulator involved in remodeling of the NMJ postsynaptic machinery. Moreover, the fact that after Tks5 knockdown other phenotypes unrelated to remodeling of cluster topology were observed indicates that this protein has multiple functions at the postsynaptic apparatus. Thus, considering that Tks5 is also an essential podosomal scaffold, chances are that its other functions can be mediated by the actin cytoskeleton [20,36]. We therefore performed PLA to check whether association of actin with rapsyn (anchoring AChRs to cortical actin filaments) was affected by the absence of Tks5. The obtained results suggest that the aberrant organization of AChR clusters in Tks5-depleted myotubes described above are likely due to the reduced number of actin sites interacting with rapsyn present at these assemblies (Figure 4). Furthermore, an analysis of actin filament-staining with fluorescent phalloidin also seems to show a decrease of the actin filament-associated fluorescence within and in the vicinity of the AChR clusters, though this certainly requires more detailed examination. For example, it would be interesting to see whether coupling of actin filaments to the postsynaptic region by Tks5 is regulated by its binding to αDB1. It has been shown that the three tyrosine phosphorylation sites located on αDB1 are important for maintaining stability of the NMJ [23]. Interestingly, the postsynaptic phenotypes observed by Grady and others in mice lacking αDB1 were similar to the ones that we described for myotubes lacking Tks5 (Figure 3) [23]. In both cases, AChR turnover was compromised, and the AChR distribution within the clusters appeared to be more diffused (Figure 3) [23]. Importantly, αDB1 constructs with mutated phosphotyrosine sites were ineffective at restoring the KO phenotypes when electroporated into the muscle [23]. This suggests that the three sites have functional significance in αDB1. Interestingly, Pawlikowski and Maimone have demonstrated that αDB1 is required for formation of complex pretzel-like AChR clusters in vitro, and that the three tyrosine phosphorylation sites of αDB1 are needed for rescuing the KO phenotype [24]. A large portion of postsynaptic machinery components is transcribed locally within the perisynaptic nuclei [37,38]. Since actin filaments are also used for a short-distance cargo delivery, the maintenance of postsynaptic specialization is highly dependent on the interaction with this cytoskeletal component. This may be the reason why we observed aberrations in the AChR turnover once Tks5 was knocked down. Since this protein seems to be needed for recruitment of actin to the postsynaptic machinery, the observed phenotypes may be an indication of compromised delivery pipeline of synaptic components.

The molecular mechanism for Tks5-driven podosome formation has been previously described in other cell types, and chances are that similar processes take place in myotubes [39] (Figure 4D). Tks5 is initially phosphorylated by non-receptor tyrosine kinase, c-SRC, and is recruited to the cell membrane by an adaptor protein GRB2, which has been also shown to interact with αDB1 in the muscle, where it is required for AChR organization [21,28,40]. Then, Tks5 binds to proteins that are essential for podosome formation, including N-WASP, which clusters at the site of adhesion and associates with actin related protein complex 2/3 (ARP2/3) [28,41]. Presence of ARP2/3 allows polymerization and branching of the actin filaments, indispensable for podosome protrusion. We believe that Tks5 could regulate podosome formation by recruitment of ARP2/3 to filaments. The discovery of the mechanism of Tks5-driven recruitment of actin to the NMJ postsynaptic machinery will be an exciting avenue of future studies, initiated by the novel observations presented herein.

## 4. Materials and Methods

### 4.1. Cell Culture

C2C12 myoblasts were acquired from American Type Culture Collection (catalog No. CRL1772, Manassas, VA, USA). Cells were cultured on 10-cm petri dishes covered with air-dried 0.2% gelatin in Dulbecco’s Modified Eagle Medium—DMEM (catalog No. D6046, SIGMA, Saint Louis, MO, USA) supplemented with 20% fetal bovine serum (FBS; catalog No. E5050-02, EURx, Gdańsk, Poland), 4.5 g/L of glucose, 2 mM of L-glutamine, 100 IU/mL of penicillin, 100 μg/mL of streptomycin and fungizone, and incubated at 37 °C and 5% CO_2_. Permanox slides (catalog No. 160005, Thermo Fisher Scientific, Waltham, MA, USA) with ethanol-washed flexiperm 8-well grids (0.9 cm^2^ surface area per well) (catalog No. 6032039, Sarstedt, Nümbrecht, Germany) attached to them were used for all experiments on in vitro cultured myotubes. To stimulate formation of complex assemblies of the postsynaptic machinery, the surface of one well on a flexiperm slide was covered with 2 μg of mouse laminin 111 (catalog No. L2020-1MG, Sigma, Saint Louis, MO, USA).

### 4.2. Cell Transfection

C2C12 myotubes were transfected using 20 nM of siRNAs, 72 h after replacing the growth medium with differentiation medium. The siRNAs were incubated with Lipofectamine RNAiMAX (catalog No. 13778075, Thermo Fisher Scientific, Waltham, MA, USA) for 10 min at room temperature. The siRNA–lipofectamine mixture was added dropwise to the designated wells and protein knock down was verified using Western Blot analysis. The cell lysis or fixation for microscopy was carried out 48 h after the addition of siRNAs. The negative control was a scrambled sequence (catalog No. 12935300, Invitrogen, Waltham, MA, USA)

### 4.3. Immunocytochemistry and Immunohistochemistry

In order to prevent unspecific binding of antibodies, fixed cells were blocked using a blocking buffer (2% BSA; catalog No. SC-2323, ChemCruz, Dallas, TX, USA]; 2% NGS; 0.5% Triton X-100 (catalog No. T8787-100ML, Sigma, Saint Louis, MO, USA), diluted in PBS). After 1 h incubation, the blocking buffer was removed and antibodies were added to the wells. If only the proteins present on the surface of the cells were to be stained, the blocking buffer did not contain Triton X-100. Primary antibodies were diluted in blocking buffer at 1:250 dilution. We added 200 μL of the antibody solution to each Flexiperm well and the cells were incubated at 4 °C overnight. The following day primary antibodies were gently aspirated and cells were washed 3 times with PBS. Secondary antibodies diluted in the blocking buffer at 1:500 ratio (1:1000 for BTX) were then added to appropriate wells and incubated for 1 h at room temperature. After that period, the antibodies were gently aspirated, and the cells were washed 3 times with PBS. For staining of muscle tissues, the fixed muscles were cut into single fibers and incubated in the blocking buffer. Antibody binding was performed using the same antibody concentrations and the same buffers as described above.

### 4.4. Microscopy and Image Processing

Microscopic analysis was performed at the Laboratory of Imaging Tissue Structure and Function, Nencki Institute, using a Zeiss Spinning Disc confocal microscope that was equipped with a diode. The objectives used were 63× and 20×. Images were collected using ZEN software (ZEISS International, Oberkochen, Germany) and quantification analyses were performed using ImageJ/Fiji software.

### 4.5. Proximity Ligation Assay (PLA)

The assay was used to visualize protein–protein interactions. Briefly, β-actin and rapsyn were labeled with primary antibodies, followed by the addition of secondary antibodies conjugated to oligonucleotide sequences. If the two proteins are in close proximity, the oligonucleotides can ligate and be further amplified with polymerase. A fluorescent probe was then added and after binding to the amplified oligonucleotides the signals were detected with a confocal microscope. The positive signals indicate that two proteins are away from each other by a distance of maximally up to 40 nm and are considered to reflect a protein–protein interaction [31].

All of the reagents (except from primary antibodies) to perform the PLA were provided in a single kit (catalog No. DUO92101, Sigma, Saint Louis, MO, USA). The assay was performed in accordance with the manufacturer’s instructions, using monoclonal anti-rapsyn (catalog No. ab156002, Cambridge, UK) and monoclonal anti-β-actin (catalog No. A1978, Sigma, Saint Louis, MO, USA) primary antibodies. The kit that was used contained anti-mouse and anti-rabbit secondary antibodies.

### 4.6. Protein Pulldown

Prior to the experiment, magnetic beads (catalog No. Dynabeads^TM^ Protein G 10004D, Invitrogen, Waltham, MA, USA) were resuspended by vortex. The total amount needed for the experiment (25 μL per sample) was transferred to an Eppendorf tube, and placed on a magnet in order to separate the beads from the buffer. The supernatant was aspirated and beads were rinsed 3 times with PBS + 0.1% NP40. 20 μg (per sample) of the appropriate αDB-1 peptide were diluted in 50 μL of PBS + 0.1% NP40 and added to the beads, followed by a 30-min incubation with rotation at room temperature. Two peptides were used in parallel during the experiment, one unphosphorylated containing the sequence around Y730 αDB-1 (LEEYLKQK), and the second one containing the same sequence with phosphorylated Y730 (LEEpYLKQK). The phosphorylated peptides were generated by incorporating protected phospho-tyrosine directly into the sequence. After removing the supernatant containing unbound peptides, the bead-peptide complex was washed 2 times with PBS + 0.1% NP40 and 1 time with the lysis buffer in order to calibrate the beads. In order to purify the target complexes, 200 μL of cell lysate (per sample) were added to the beads-peptide complex, followed by an overnight incubation with rotation at 4 °C. The next day, tube containing beads–peptide–protein complexes was placed on a magnet and the supernatant (flow-through) was removed and stored to be used as control later on. The beads were then washed 4 times with the lysis buffer. Once the last wash was discarded from the tube containing the beads–peptide–protein complex, it was removed from the magnet and the beads were resuspended in 30 μL (per sample) of 2×Laemmli Sample Buffer + 50 mM DTT, followed by a 10-min incubation at 95 °C. After denaturation the samples were immediately loaded on the gel, or frozen in −20 °C.

### 4.7. Mass Spectrometry

The mass spectrometry analysis was carried out in order to find interacting partners of αDB1, which bind to it either when the tyrosine-730 is phosphorylated or unphosphorylated. For this purpose, the mass spectrometry analysis was performed on eluates obtained by coupling the phosphorylated and unphosphorylated DB1-730 peptides to Dynabeads M-270 and incubation with C2C12 cell lysates. A combined lysate from ten 15-cm plates was used per sample. The whole procedure was carried out at 4 °C. The growth medium was aspirated and each plate containing cells was first gently washed with 20 mL of cold PBS in order to remove any remaining medium. Then, 1.5 mL of lysis buffer were then added per plate and the cells were scraped in order to facilitate the lysis. The lysate from all plates was collected into a single 50-mL tube. The lysate was then passed through a 25-gauge needle 5 times followed by rotation for 30 min in the cold room. Next the lysate was centrifuged at 18,000× *g* at 4 °C for 30 min and the supernatant was placed in a fresh, chilled 50-mL tube. For each sample, 440 μL of Epoxy M-270 beads (catalog No. 14301, Invitrogen, Waltham, MA, USA) were used. The beads were first placed on a magnet and the supernatant was removed, followed by 4 washes with PBS + pervanadate (PVD). Then, 40 μL of either phosphorylated DB1-730 or unphosphorylated DB1-730 were added to the beads followed by a 30-min incubation at room temperature. After that, the tubes were placed on a magnet, supernatant was removed, and the samples were washed 3 times with PBS + PVD and 2 times with the lysis buffer. Once the beads coupled with appropriate peptides were washed, they were added to C2C12 lysates, followed by an overnight incubation with rotation at 4 °C. Following the overnight incubation, the tubes were placed on a magnet, supernatant was collected and beads were washed 3 times with the lysis buffer. Then, 40 μL of 2× Sample Buffer + DTT were used to resuspend each sample. The samples were then placed on a heat block and incubated for 10 min at 95 °C. The mass spec analysis was outsourced to the MS Bioworks facility where protein samples were analysed with 0.5 h LC-MS/MS and protein identification was performed with Mascot.

### 4.8. Mice Sacrifice and Tissue Extraction

Wild-type C57BL P7 and P60 male mice were used. Mice were sacrificed by placing them on cotton pads soaked with 1000 mg/g of isoflurane inside a beaker. To extract tibialis anterior, the leg was cut off and skin together with fascia were removed. The procedures were conducted in accordance with all applicable laws and regulations.

### 4.9. Muscle Electroporation

The electroporation was performed on mice anesthetized with ketamine (150 mg/kg)/xylazine (10 mg/kg) cocktail, each time injecting 25 µg of plasmid DNA. After plasmid injection, the TA muscle was electroporated with 10 pulses of 180 V/cm current at 1 s interval between the consecutive pulses. For the procedure the ECM-830 electroporator (catalog No. 45-0052INT, BTX Harvard Apparatus, Holliston, MA, USA) was used. The experiment was repeated three times, each time using a new mouse so in total three P7 and three P60 mice were used. All mice were male. approved by the Ethics Committee of Nencki Institute of Experimental Biology (protocol code 629/2018 22.05.2018).

## Data Availability

The datasets generated or analyzed during the current study are available from the corresponding author upon a reasonable request.
